# Changes in metamorphopsia after the treat-and-extend regimen of anti-VEGF therapy for macular edema associated with branch retinal vein occlusion

**DOI:** 10.1371/journal.pone.0241343

**Published:** 2020-10-28

**Authors:** Kenichiro Mori, Keijiro Ishikawa, Iori Wada, Yuki Kubo, Yoshiyuki Kobayashi, Takahito Nakama, Masatoshi Haruta, Masato Akiyama, Shintaro Nakao, Shigeo Yoshida, Koh-Hei Sonoda

**Affiliations:** 1 Department of Ophthalmology, Graduate School of Medical Sciences, Kyushu University, Fukuoka, Japan; 2 Department of Ophthalmology, Graduate School of Medical Sciences, Kurume University, Kurume, Japan; National Yang-Ming University Hospital, TAIWAN

## Abstract

This study aims to investigate the changes in metamorphopsia after administering the treat-and-extend regimen of anti-vascular endothelial growth factor therapy for branch retinal vein occlusion-associated macular edema. We retrospectively examined 27 patients (27 eyes) with macula edema due to branch retinal vein occlusion who received intravitreal injections of anti-vascular endothelial growth factor agents using the treat-and-extend regimen for ≥18 months. We evaluated best-corrected visual acuity, central macular thickness, macular edema recurrence, and amount of metamorphopsia quantified by M-CHARTS. The best-corrected visual acuity (logarithm of minimum angle of resolution) and central macular thickness significantly improved at 18 months compared to baseline, the median value (interquartile range [IQR]), 0.30 (0.15–0.52) and 459 (373–542) μm at baseline, and 0 (-0.08–0.16) and 267 (232–306) μm at 18 months. The M-CHARTS score (the mean of vertical and horizontal scores) significantly decreased at 1, 6, and 12 months compared to baseline, but worsened at 18 month, the median value (IQR), 0.45 (0.250–0.925), 0.4 (0.15–0.70), 0.4 (0.150–0.625), 0.4 (0.225–0.550) and 0.45 (0.225–0.750) at baseline, 1 month, 6 months, 12 months and 18 months, respectively. The median cumulative number of macular edema recurrences was 2 (IQR, 0.5–3.0) at 18 months. Simple linear regression and multivariate analyses revealed that the change in the mean M-CHARTS score at 18 months was significantly correlated with the baseline score and the cumulative number of macular edema recurrences. Anti-vascular endothelial growth factor therapy using the treat-and-extend regimen improved metamorphopsia in branch retinal vein occlusion-related macular edema in the short to mid-term follow-up period, but not in the long term. Macular edema recurrence may be associated with persistent metamorphopsia.

## Introduction

Branch retinal vein occlusion (BRVO) is one of the most common retinal vascular diseases. Macular edema (ME) secondary to BRVO is a major complication and decreases visual acuity (VA) [1, 2]. Randomized clinical trials regarding BRVO, such as BRIGHTER, VIBRANT, and BRAVO revealed that intravitreal injections of anti-vascular endothelial growth factor (VEGF) agents substantially improved ME and VA [3–5]. However, BRVO patients often have decreased quality of vision due to symptomatic metamorphopsia, even after VA improvement and ME resolution [6–9]. It has been reported that improvement of metamorphopsia was more strongly associated with good vision-related quality of life than VA restoration in patients with an epiretinal membrane and a macular hole who underwent vitrectomy [10]. Therefore, clinicians need to strongly consider metamorphopsia improvement to increase the quality of life of patients with vitreoretinal disorders.

After anti-VEGF therapy for ME in BRVO, metamorphopsia improves in the short term (1 month) [7, 8], but not in the midterm (6–12 months) despite ME improvement [8, 9], and the long term (>12 months) outcome has not been reported. In previous reports, patients were treated with intravitreal injections of anti-VEGF agents using a pro re nata (PRN) regimen. To date, no report has studied the change in metamorphopsia after anti-VEGF therapy using the treat-and-extend regimen (TAE) regimen. This study aimed to determine the effects of the TAE regimen of anti-VEGF therapy on metamorphopsia in patients with BRVO during an 18-month follow-up.

## Methods

### Patients

We retrospectively reviewed the medical records of 27 patients (27 eyes) with ME secondary to BRVO. All the eyes were treated with intravitreal injections of anti-VEGF agents (ranibizumab or aflibercept) using a TAE regimen for at least 18 months at Kyushu University Hospital between April 2014 and April 2017.

The inclusion criteria were (1) symptomatic BRVO with retinal hemorrhage and edema involving the macula and (2) central macular thickness (CMT) greater than 300 μm measured by optical coherence tomography (OCT) at baseline. We diagnosed BRVO based on fundus examinations and fluorescein angiography findings.

The exclusion criteria were (1) history of vitreoretinal surgery and treatment with anti-VEGF agents, (2) previous treatment with sub-Tenon triamcinolone acetonide injection within 3 months, (3) uncontrolled glaucoma, (4) active intraocular inflammation, and (5) central retinal vein occlusion or hemi-central retinal vein occlusion.

Our study was approved by the Institutional Ethics Committee of Kyushu University Hospital (Fukuoka, Japan) and was conducted according to the principles of the Declaration of Helsinki on human biomedical research. All patients provided written informed consent to participate in our study.

### Ophthalmological examination

At each follow-up visit, the examinations included measurements of best-corrected VA and amount of metamorphopsia, which was quantified using M-CHARTS, slit-lamp biomicroscopy, and OCT. We assessed best-corrected VA using the Landolt decimal VA charts (CV-6000, Tomey, Nagoya, Japan) or single Landolt test cards (HP-1258, Handaya, Tokyo), and best-corrected VA was expressed as the logarithm of the minimal angle of resolution (logMAR). OCT examination was performed using CIRRUS HD-OCT 5000 (Carl Zeiss, Meditec, Dublin, CA). We used a macular thickness map protocol with a macular cube of 512 × 128. The mean thickness within the central 1-mm diameter circle was defined as the CMT.

### M-CHARTS

We evaluated the amount of metamorphopsia, which was quantified using M-CHARTS. The principle behind M-CHARTS has been described in previous reports [11–13]. The M-CHARTS examination is composed of a series of 19 dotted line tests, with dot intervals ranging from 0.2° to 2.0°. The metamorphopsia of the line decreases with the increasing dot interval. First, we showed a vertical straight line to the patient at a distance of 30 cm with the refraction of the eye corrected. Next, we examined with vertical lines. If the patient recognized a straight line as distorted, then we showed the following pages with an increasing dot interval until the dotted line seemed straight. When the patient recognized the dotted line as straight, the visual angle of that line was determined to represent their metamorphopsia score for vertical lines. Thereafter, the M-CHARTS were rotated 90° and we repeated the test with horizontal lines. Vertical and horizontal scores were independently obtained and their mean scores were calculated. The change in the mean M-CHARTS score at 18 months after treatment was defined as the mean M-CHARTS score at 18 months minus the mean M-CHARTS score at baseline.

### Treat-and-extend regimen

All patients were treated with intravitreal injections of anti-VEGF agents using a TAE regimen. The intravitreal dose of ranibizumab was 0.5 mg/0.05 mL, and that of aflibercept was 2 mg/0.05 mL. The patients received injections of anti-VEGF agents every 4 weeks until an improvement of ME was confirmed by OCT. ME recurrence was defined as a CMT > 300 μm during the treatment course. If there was no ME recurrence, a new injection was given, and the injection interval was extended by 2 weeks to a maximum interval of 12 weeks. If there was a ME recurrence, the injection interval was shortened by 2 weeks to a minimum of 4 weeks. Patients whose injection interval was extended to 12 weeks did not receive an additional injection and were subsequently switched from a TAE to a PRN regimen. Those patients were followed up 1 month later. If they had no ME, they were followed up every 12 weeks.

### Statistical analysis

Statistical analyses were performed using a commercial statistical software package (JMP, ver. 12.2.0; SAS Institute, Cary, NC, USA). All values are presented as the median (interquartile range [IQR]). The Wilcoxon signed-rank test was used to compare logMAR VA, CMT, and the vertical, horizontal, and mean M-CHARTS scores. Bivariate relationships were examined using simple linear regression analysis to evaluate the factors associated with the mean M-CHARTS score and its change at baseline and 18 months after treatment. To assess independently associated factors, we selected variables which were significantly associated in the simple linear regression analyses (P < 0.05), and performed a multiple linear regression analysis using the selected variables after confirming that these variables were not significantly correlated (P for Pearson’s correlation > 0.05) in order to avoid multicollinearity. Statistical significance was defined as P < 0.05.

## Results

**Fig 1:**
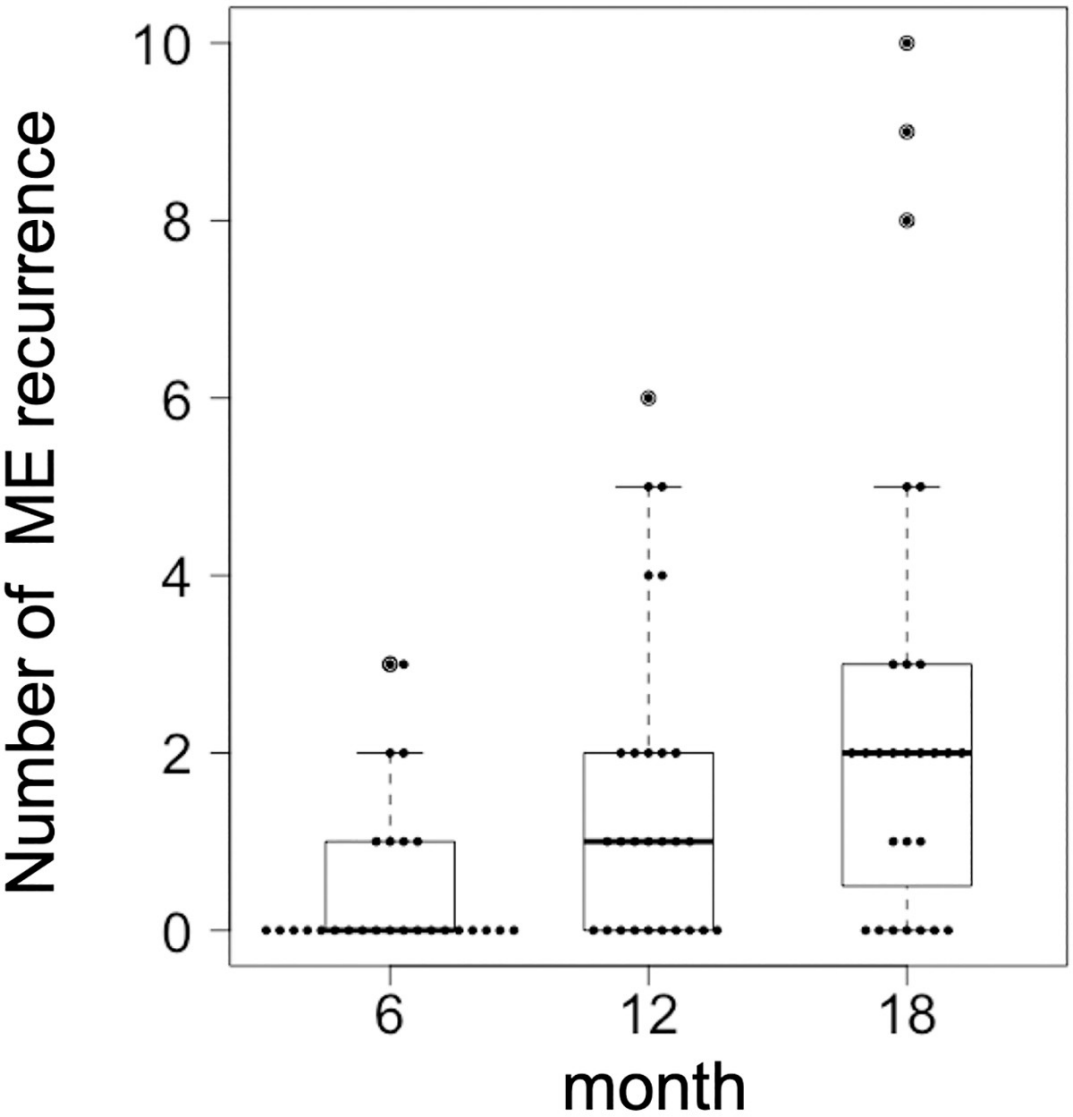
Cumulative number of macular edema recurrences after treatment. The cumulative numbers of macular edema recurrences at 6, 12, and 18 months after treatment are visualized by dot in box-and-whisker plots. The thick horizontal line within each box indicates the median score, and the top and bottom of each box indicate the 75th and 25th percentiles. Whiskers above the box indicate values within 1.5× of the interquartile range (IQR) of the upper quartile, and whiskers below the box indicate values within 1.5 × of the IQR of the lower quartile. ME = macular edema.

The baseline characteristics of the patients are shown in Table 1. Twenty-three (85.2%) of 27 eyes had metamorphopsia at baseline. The median number of injections was 7 (IQR, 5–7) up to 12 months and 8 (IQR, 6–9) up to 18 months. ME recurrence occurred in 20 eyes (74.0%) during the follow-up period. The median cumulative number of ME recurrences was 0 (IQR, 0–1), 1 (IQR, 0–2), and 2 (IQR, 0.5–3) at 6, 12, and 18 months after treatment, respectively (Fig 1). Fifteen patients were switched to the PRN regimen during the follow-up period.

**Table 1:** Baseline characteristics of eyes with branch retinal vein occlusion.

Sex (Male/Female)	10/17
Age (years)	71 (62.5–75)
Duration of symptoms (weeks)	10 (5.5–18.5)
LogMAR visual acuity	0.30 (0.15–0.52)
CMT (μm)	459 (373–542)
M-CHARTS score	
Vertical	0.5 (0.1–1.25)
Horizontal	0.5 (0.2–0.6)
Mean	0.45 (0.25–0.925)
SRD (eyes (%))	10 (37)
Hypertension (eyes (%))	13 (48)

All values are presented as the median (IQR).

logMAR = logarithm of the minimum angle of resolution; CMT = central macular thickness; SRD = serous retinal detachment.

**Fig 2:**
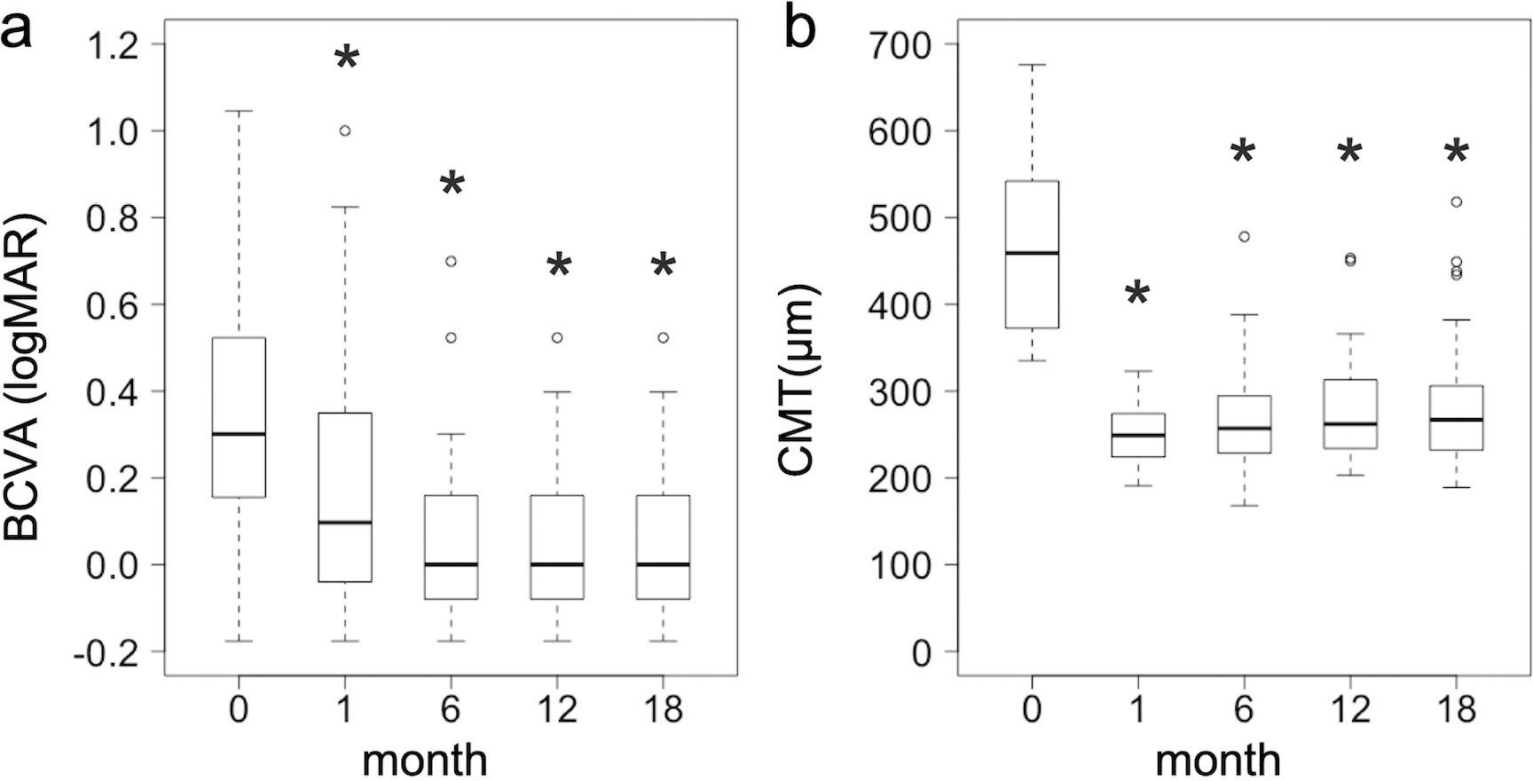
LogMAR VA and CMT changes after treatment. LogMAR VA (a) and CMT (b) changes at 1, 6, 12, and 18 months after treatment (n = 27) are visualized by box-and-whisker plots. The thick horizontal line within each box indicates the median score, and the top and bottom of each box indicate the 75th and 25th percentiles. Whiskers above the box indicate values within 1.5× of the interquartile range (IQR) of the upper quartile, and whiskers below the box indicate values within 1.5× of the IQR of the lower quartile. Best-corrected VA (logMAR) = best-corrected visual acuity (logarithm of minimal angle of resolution); CMT = central macular thickness. * Indicates P < 0.0001 compared to baseline values.

**Fig 3:**
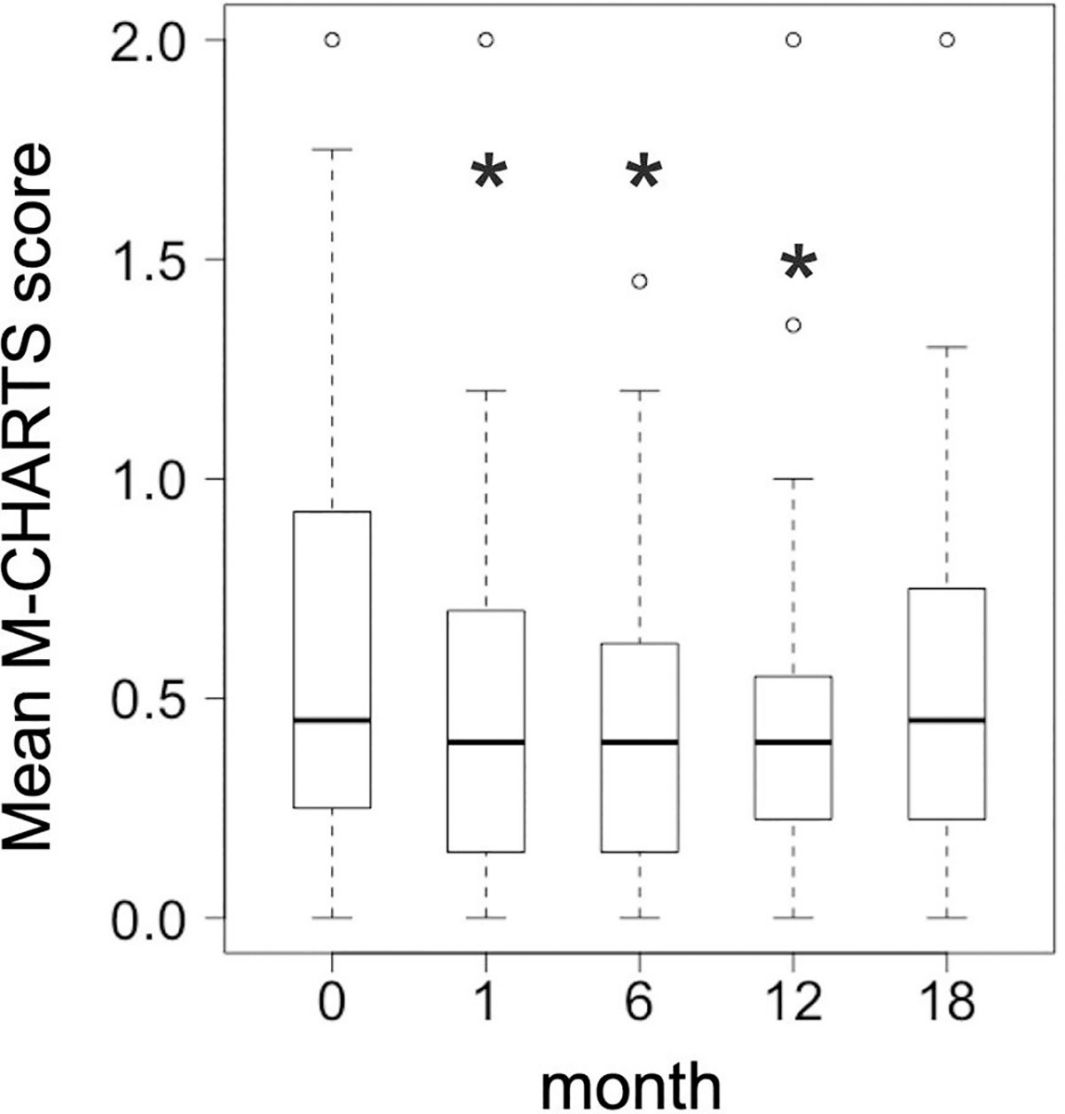
Changes in the mean metamorphopsia score after treatment. Changes in the mean metamorphopsia scores (the mean values of the vertical and horizontal scores) at 1, 6, 12, and 18 months after treatment (n = 27) are visualized by box-and-whisker plots. The thick horizontal line within each box indicates the median score, and the top and bottom of each box indicate the 75th and 25th percentiles. Whiskers above the box indicate values within 1.5× of the interquartile range (IQR) of the upper quartile, and whiskers below the box indicate values within 1.5× of the IQR of the lower quartile. * Indicates P < 0.05 compared to baseline values.

**Fig 4:**
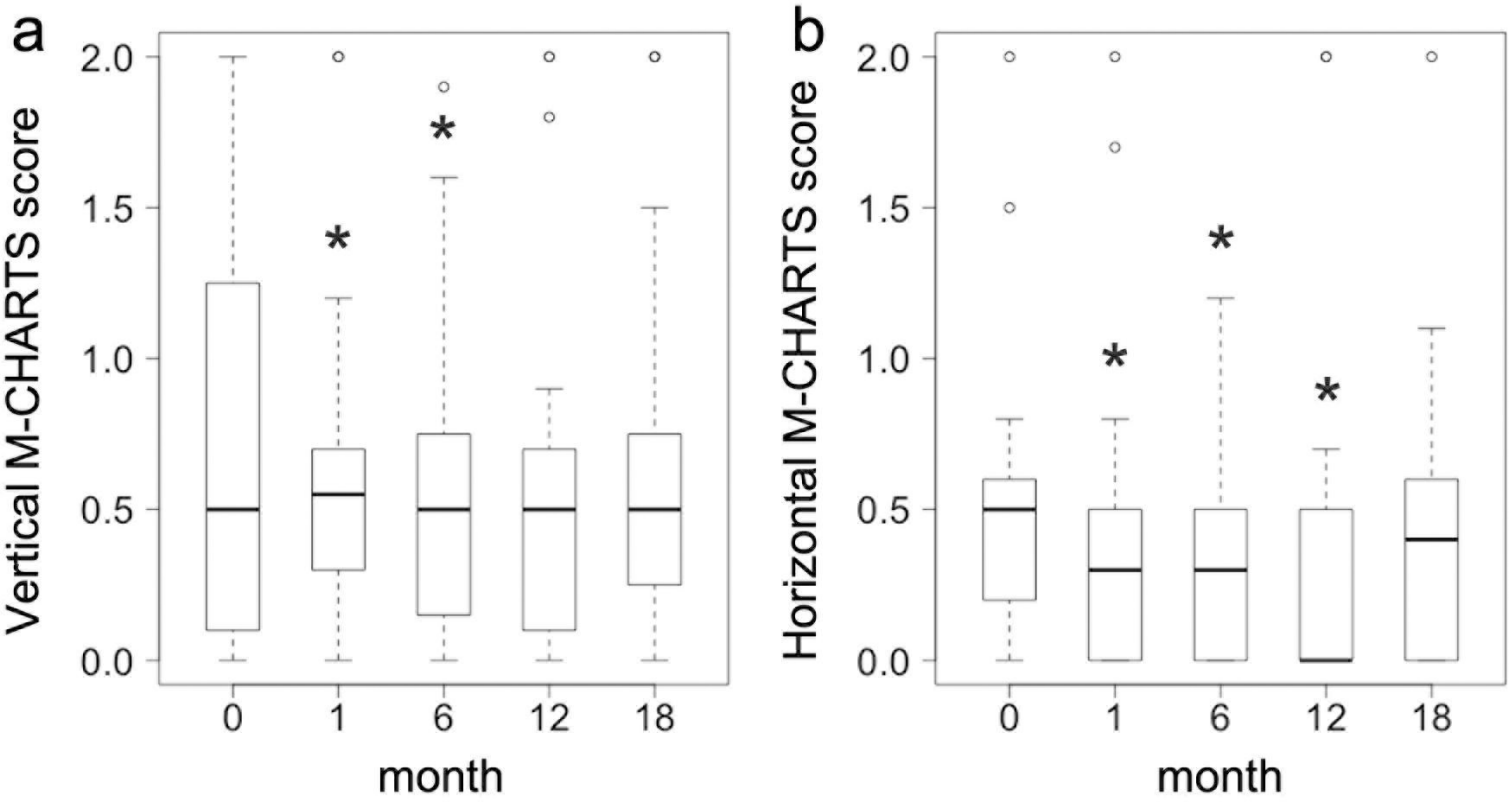
Changes in the vertical and horizontal metamorphopsia scores after treatment. Changes in the vertical (a) and horizontal (b) metamorphopsia scores at 1, 6, 12, and 18 months after treatment (n = 27) are visualized by box-and-whisker plots. The thick horizontal line within each box indicates the median score, and the top and bottom of each box indicate the 75th and 25th percentiles. Whiskers above the box indicate values within 1.5× of the interquartile range (IQR) of the upper quartile, and whiskers below the box indicate values within 1.5× of the IQR of the lower quartile. * Indicates P < 0.05 compared to baseline values.

The best-corrected VA and CMT significantly improved at 18 months compared to baseline, the median value (interquartile range [IQR]), 0.30 (0.15–0.52) and 459 (373–542) μm at baseline, and 0 (-0.08–0.16) and 267 (232–306) μm at 18 months (P < 0.0001) (Table 2 and Fig 2). The mean M-CHARTS score significantly improved at 1, 6, and 12 months compared to baseline (P = 0.005, P = 0.015, and P = 0.006, respectively), but the score at 18 months was not significantly different (P = 0.080) (Table 2 and Fig 3). The vertical M-CHARTS scores significantly improved at 1 and 6 months and the horizontal M-CHARTS scores significantly improved at 1, 6 and 12 months compared to baseline. The score changes were not significantly different at 18 months as noted with the mean score change (Table 2 and Fig 4).

**Table 2:** Changes in ocular parameters after treatment for macular edema associated with branch retinal vein occlusion.

	Baseline	1 month	6 month	12 month	18month
BCVA (logMAR)	0.30	0.10	0	0	0
	(0.15–0.52)	(-0.04–0.35)^†^	(-0.08–0.16)^†^	(-0.08–0.16)^†^	(-0.08–0.16)^†^
CMT (μm)	459	249	257	262	267
	(373–542)	(224–274)^†^	(229–295)^†^	(234–313)^†^	(232–306)^†^
M-CHARTS score					
Vertical	0.5	0.55	0.5	0.5	0.5
	(0.1–1.25)	(0.3–0.7)*	(0.15–0.75)*	(0.1–0.7)	(0.25–0.75)
Horizontal	0.5	0.3	0.3	0	0.4
	(0.2–0.6)	(0–0.5)*	(0–0.5)*	(0–0.5)*	(0–0.6)
Mean	0.45	0.4	0.4	0.4	0.45
	(0.25–0.925)	(0.15–0.7)*	(0.15–0.625)*	(0.225–0.55)*	(0.225–0.75)

BCVA (logMAR) = best-corrected visual acuity (logarithm of minimal angle of resolution); CMT = central macular thickness.

^†^ Wilcoxon signed-rank test compared to baseline values (P < 0.0001)

* Wilcoxon signed-rank test compared to baseline values (P < 0.05).

S1 and S2 Tables show the association between the mean M-CHARTS score and parameters at baseline and at 18 months after treatment, respectively. A simple linear regression analysis regression analysis revealed a significant correlation between the mean M-CHARTS score and BCVA at 18 months after treatment (β = 1.350, P = 0.004). However, there was no correlation between the mean M-CHARTS score and characteristics at baseline.

Table 3 shows the association between the change in the mean M-CHARTS score at 18 months after treatment and baseline parameters, and the number of ME recurrences. Simple linear regression analysis revealed that the mean M-CHARTS score change was significantly correlated with the baseline mean M-CHARTS score (β = -0.649, P < 0.001) and the number of ME recurrences (β = 0.097, P = 0.01). Multiple linear regression analysis revealed that the change in the mean M-CHARTS score was significantly correlated with the baseline mean M-CHARTS score (β = -0.572, P < 0.001) and the number of ME recurrences (β = 0.073, P = 0.02).

**Table 3:** Association between the change in the mean M-CHARTS score at 18 months after treatment and baseline parameters and number of macular edema recurrence.

	Simple linear regression analysis			Multiple linear regression analysis		
	β	SE	P-value	β	SE	P-value
Baseline age	0.017	0.012	0.18			
Baseline BCVA (logMAR)	0.339	0.376	0.38			
Baseline CMT	-0.0001	0.001	0.90			
Baseline MM	-0.649	0.156	< 0.001	-0.572	0.144	< 0.001
Baseline SRD	-0.083	0.111	0.46			
Number of ME recurrence	0.097	0.036	0.01	0.073	0.029	0.02

β = regression coefficient; SE = standard error; BCVA (logMAR) = best-corrected visual acuity (logarithm of minimal angle of resolution); CMT = central macular thickness; MM = mean M-CHARTS score among vertical and horizontal; SRD = serous retinal detachment; ME = macular edema.

## Discussion

Metamorphopsia is present in the majority of patients (68.8–93%) with BRVO [6–9] as noted in the present study (85.2%). Some studies about changes in metamorphopsia after anti-VEGF therapy with PRN regimen for BRVO-ME using M-CHARTS showed that the scores were not significantly decreased in the short term up to 6 months after induction even with ME improvement [6, 7, 9]. Osaka et al. reported significant improvement in M-CHARTS scores at 1 month; however, the score worsened with no significant improvement at 12 months compared to baseline, and the mean cumulative number of ME recurrences was 3 ± 1.8 [8]. In contrast, our data show the improvement of the metamorphopsia with significant decrease of the mean M-CHARTS score was maintained up to 12 months, and the mean cumulative numbers of ME recurrences was 1.5 ± 1.8 (median: 1 [IQR, 0–2]). These observations suggest that our proactive TAE regimen could reduce the frequency of ME recurrence leading to sustained improvement of metamorphopsia after anti-VEGF therapy induction during the short to middle term.

On the other hand, the mean M-CHARTS score gradually increased after 12 months but was eventually not significantly different at 18 months compared with the baseline score. The mean cumulative number of ME recurrences at 18 months (2.5 ± 2.7, median: 2 [IQR, 0.5–3]) was comparable to the number at 12 months reported in a previous study with a PRN regimen, in which a significant M-CHARTS score improvement was not found [8]. The significantly positive relationship between the change in the mean M-CHARTS score and the cumulative number of ME recurrences in our study further suggest that the increase in the number of cumulative recurrences could cause prolonged ME, resulting in posttreatment metamorphopsia even after the achievement of improved VA and ME resolution. This observation is supported by a previous finding where posttreatment metamorphopsia was associated with the duration of symptoms before initiating anti-VEGF therapy for BRVO-ME [9]. Since long-term retinal morphological abnormalities including ME can lead to irreversible photoreceptor cell damage and changes in cone-cell alignment [14, 15], preventing ME recurrence to shorten ME duration might be important to maintain metamorphopsia improvement during anti-VEGF treatment for patients with BRVO-ME. Our present study showed no correlation between M-CHARTS scores and CMT at baseline and 18 months, which is not consistent with previous findings of a significant correlation between them at baseline [7]. This discrepancy could be due to the longer duration of symptoms before treatment in our study compared to the previous study (10 weeks vs. 5.5 weeks), because long-term retinal morphological abnormalities can lead to changes in cone cell alignment [14, 15]. The positive correlation between the mean M-CHARTS score and BCVA at 18 months suggests that eyes with better BCVA may have less photoreceptor damage during the treatment course, resulting in less metamorphopsia.

Therefore, we suggest that the TAE regimen, which is a proactive treatment, may have the advantage of improving the metamorphopsia associated with BRVO-related ME by reducing the frequency of recurrence. To maintain the metamorphopsia improvement for a longer time, it may be necessary to modify our TAE regimen to a more aggressive treatment. This could include instituting maximum treatment intervals greater than 12 weeks or prolonging treatment with a 12-week interval for a longer period, as done for the TAE regimen used in patients with age-related macular degeneration [16]. A previous study demonstrated the mean number of injections within 12, 18, and 24 months was significantly higher in the TAE regimen than in the PRN regimen in the treatments for retinal vein occlusion patients [17]. In fact, the mean number of injections within 12 months (6.6 ± 1.7, median: 7 [IQR, 5–7]) in our TAE regimen was higher than the mean number of injections within 12 months (4.0 ± 1.8) reported in the previous study with the PRN regimen [8]. Further study is needed to optimize the TAE regimen in order to achieve a sustainable quality of vision for a longer time while lowering the patient, physician, and economic burden of BRVO-associated ME.

The limitations of this study are its relatively small sample size, the use of two different anti-VEGF agents, and the lack of a comparison of the two treatment regimens. To demonstrate the advantage of the TAE regimen for metamorphopsia improvement, it is necessary to design large-scale prospective studies comparing the TAE and PRN regimens using unified anti-VEGF agents.

In conclusion, anti-VEGF therapy using the TAE regimen improved metamorphopsia in BRVO-related ME during the short to mid-term follow-up period, but not during the long-term follow-up. The number of ME recurrences may be associated with persistent metamorphopsia.

## Supporting information

S1 Table Association between the mean M-CHARTS score and parameters at baseline. (DOCX)

S2 Table Association between the mean M-CHARTS score and parameters at 18 months after treatment. (DOCX)
