# Supplementary material for: Changes in metamorphopsia after the treat-and-extend regimen of anti-VEGF therapy for macular edema associated with branch retinal vein occlusion
Source: PLoS One. 2020 Oct 28;15(10):e0241343. doi: 10.1371/journal.pone.0241343 (PMC7592807; doi:10.1371/journal.pone.0241343)
Supplement: S1 Table — (DOCX) [file pone.0241343.s001.docx]

**S1 Table. Association between the mean M-CHARTS score and parameters at baseline.**

|  | Simple linear regression analysis | | |
| --- | --- | --- | --- |
| Parameters  (baseline) | β | SE | P-value |
| Age | -0.011 | 0.012 | 0.36 |
| BCVA (logMAR) | 0.361 | 0.369 | 0.34 |
| CMT | -0.000 | 0.001 | 0.94 |
| SRD | 0.018 | 0.111 | 0.87 |

β = regression coefficient; SE = standard error; BCVA (logMAR) = best-corrected visual acuity (logarithm of minimal angle of resolution); CMT = central macular thickness; SRD = serous retinal detachment
