# Supplementary material for: Changes in metamorphopsia after the treat-and-extend regimen of anti-VEGF therapy for macular edema associated with branch retinal vein occlusion
Source: PLoS One. 2020 Oct 28;15(10):e0241343. doi: 10.1371/journal.pone.0241343 (PMC7592807; doi:10.1371/journal.pone.0241343)
Supplement: S2 Table — (DOCX) [file pone.0241343.s002.docx]

**S2 Table. Association between the mean M-CHARTS score and parameters at 18 months after treatment.**

|  | Simple linear regression analysis | | |
| --- | --- | --- | --- |
| Parameters  (18 month) | β | SE | P-value |
| Age | 0.006 | 0.010 | 0.60 |
| BCVA (logMAR) | 1.350 | 0.432 | 0.004 |
| CMT | 0.001 | 0.001 | 0.60 |
| Number of ME recurrence | 0.056 | 0.032 | 0.10 |

β = regression coefficient; SE = standard error; BCVA (logMAR) = best-corrected visual acuity (logarithm of minimal angle of resolution); CMT = central macular thickness; ME = macular edema
